# On the Electroanalytical Detection of Zn Ions by a Novel Schiff Base Ligand-SPCE Sensor

**DOI:** 10.3390/s22030900

**Published:** 2022-01-25

**Authors:** Viviana Bressi, Zahra Akbari, Morteza Montazerozohori, Angelo Ferlazzo, Daniela Iannazzo, Claudia Espro, Giovanni Neri

**Affiliations:** 1 Department of Engineering, University of Messina, I-98166 Messina, Italy; vibressi@unime.it (V.B.); zakbari@unime.it (Z.A.); angelo.ferlazzo@unime.it (A.F.); daniela.iannazzo@unime.it (D.I.); 2 Department of Chemistry, Yasouj University, Yasouj 7591874831, Iran; mmzohori@mail.yu.ac.ir

**Keywords:** Schiff base, fluorescence, electrochemical sensors, heavy metals, trace analysis of pollutants and contaminants

## Abstract

A novel bidentate Schiff base (L) is here proposed for the detection of Zn ions in water. The structure of the synthesized Schiff base L was characterized by FT-IR, ^1^H NMR and ^13^C NMR. Optical characteristics were addressed by UV-Visible spectroscopy and Photoluminescence (PL) measurements. PL demonstrated that L displays a “turn-off” type fluorescence quenching in the presence of Zn^2+^ ion in aqueous solution, indicating its ability to preferentially coordinate this ion. Based on these findings, an L-M (where M is a suitable membrane) modified screen-printed carbon electrode (SPCE) was developed to evaluate the electrochemical behavior of the Schiff base (L) with the final objective of undertaking the electroanalytical determination of Zn ions in water. Using various electrochemical techniques, the modified L-M/SPCE sensor demonstrates high sensitivity and selectivity to Zn ions over some common interferents ions, such as Ca^2+^, Mg^2+^, K^+^, Ni^++^ and Cd^++^. The potentiometric response of the L-M/SPCE sensor to Zn ions was found to be linear over a relatively wide concentration range from 1 μM to 100 mM.

## 1. Introduction

Schiff base ligands have been known about for a very long time and can be easily synthesized by condensation reactions of primary amines and carbonyl compounds in which the characteristic azomethine bond is formed [1]. Interest in Schiff bases has rapidly grown, and they played a central role in the development of coordination chemistry due to their unique properties, including high stability and selectivity to metal coordination, good reproducibility, more active sites and good homogeneity, making them particularly attractive in the field of metal ion recognition, especially by means of optical techniques [2,3,4].

In recent decades, electrochemical sensors have received great attention due to their good versatility in heavy metal ion detection. Several Schiff bases have been used in the development of innovative electrodes for the electrochemical recognition of heavy metal ions in water [5,6,7].

The greatest interest now relates to heavy metals such as lead, mercury, and cadmium, which even at very low concentrations can be harmful to human health. These metals are introduced into the aquatic ecosystem and the soil because of the erosion of rocks, volcanic eruptions, or by multiple human activities, such as production of industrial waste. Among the various heavy metal ions, Zn^2+^ plays an important role in the regulation of many reactions catalyzed by enzymes in the human body and therefore maintains physiological processes at the optimal level. Zinc is considered as an essential trace element for human beings due to its relationship with insulin production [8,9], and for the regulation of biological processes, being involved as catalysts for more than 200 enzymes [10]. In addition, zinc is also present in food, e.g., proteins that are high in zinc content. Although Zn^2+^ is considered relatively non-toxic, at high concentration, symptoms of toxicity can manifest such as nausea, vomiting, lethargy and fatigue [11]. Recent studies have shown that the possible alteration of serum levels of Zn^2+^ is related to the development of neurodegenerative pathologies, in particular, Alzheimer’s disease [12,13]. This cation is also presents in waste products from different industries such as paint, electroplating, pharmaceutical, and fine chemical manufacturing. For this reason, zinc ion detection is highly demanded.

Conventional methods employed for contaminant monitoring suffer from several drawbacks, requiring expensive devices and complex sample handling, in addition to being time-consuming and highly destructive [14]. Among the several kinds of electrodes, mercury-based electrodes have been traditionally used in the stripping techniques due to their high sensitivity. Hence, they have widely been accepted as the most sensitive electrodes for the determination of heavy metals. Moreover, different electrochemical techniques have been used to detect and measure Zn ions using mercury-based electrodes [15,16,17,18].

However, the use of these techniques has tended to decrease because the use of mercury affects the environment. As the use of mercury becomes increasingly restricted by health and safety regulations, the development of electrochemical approaches with high selectivity and sensitivity for zinc, that do not involve the use of mercury-based electrodes, is currently of great interest as they allow prompt detection of metal ions by simple responses. In particular, the development of electrochemical sensors that can discriminate Zn^2+^ from the elements of the same group of the periodic table with similar properties is still a challenge. Our approach fits into this scientific and technological context with the aims to identify and develop an innovative electrochemical method for the determination of zinc, by using a screen-printed electrode modified with a novel bidentate Schiff base named L. The synthesis and complexation of the base Schiff ligand L for preparing ZnL complexes and their sensing properties have been reported in a previous work [19]. Considering the peculiar characteristics of this Schiff base, we initiated a study to investigate this ligand as a sensing element for developing a new electrochemical sensor for Zn ions.

## 2. Materials and Methods

### 2.1. Chemicals

Starting reagents for the synthesis of Schiff base ligand L were purchased from commercial suppliers (Sigma-Aldrich Handels Gmbh, Overijse, Belgium) in high purity and used without further purification. Standard solution of Zn^2+^ was prepared by dissolving zinc (II) nitrate hexahydrate (0.0297 gr, 10 mM) in 10 mL of double distilled water. Similarly, all the stock solution was prepared using nitrate salts of respective cations and dissolving them in double distilled water. All chemicals were extra pure analytical grade.

### 2.2. Synthesis of Bidentate Schiff Base Ligand (L)

The Schiff base L (whose chemical name is: N1,N3-bis((E)-3-(4-methoxyphenyl)allylidene)-2,2-dimethylpropane-1,3-diamine) was synthesized under ultrasonic irradiation as follows. An ethanolic solution of 2,2-dimethylpropane-1,3-diamine (1 mmol in 10 mL) was drop-wise added to an ethanolic solution of (E)-3-(4-methoxyphenyl) acrylaldehyde (2 mmol in 10 mL). The reaction progress was monitored by TLC. After completion of the condensation reaction (30 min), the product was obtained as a milky precipitate. After evaporation of solvent, the ligand was obtained. Yield: 49%. m.p: 149–151 °C. UV–Vis in DMF [λmax; nm (λ; cm^−1^M^−1^)]: 318 (99,164), 380 (70,958). Molar conductivities in DMF [Λ°M; cm^2^ Ω^−1^ mol^−1^]: 1.12. Selected FT-IR data (KBr, cm^−1^): 3058 (ν C–H aromatic), 3014 (ν C–H alkenic), 2965 (ν C–H aliphatic), 2868 (ν C–H iminic), 1632 (ν C=N), 1462 (ν C=C), 1171 (ν C–N). 1H NMR (DMSO-d6): [δ; ppm]: 8.02(s, 2Hc,c′, J = 8.7 Hz), 7.57(d, 4Hf,f′, J = 8.87 Hz), 7.03(d, 2He,e′, J = 16.0 Hz), 6.97(d, 4Hg,g′, J = 8.8 Hz), 6.85(dd, 2Hd,d′, J1 = 16.0 Hz and, J2 = 8.7 Hz), 3.80(s,6Hh,h′), 3.35(s, 4Hb,b′), 0.92(s, 6Ha,a′) ppm. 13C NMR (DMSO-d6): 163.57 (C4,4′), 160.48 (C10,10′), 141.46 (C6,6′), 129.26 (C8,8′),128.79 (C7,7′), 126.42 (C9,9′), 114.76 (C5,5′), 70.40 (C3,3′), 55.69 (C11,11′), 37.00 (C1,1′), 24.79 (C2,2′) ppm.

### 2.3. Characterization

The FT-IR spectra (as KBr disks, 4000–400 cm^−1^) were recorded using a JASCO-460 instrument with scanning speed of 2 mm/s, resolution 4 cm^−1^, accumulation of 25 and gain of 2. The electronic spectrum of the Schiff base ligand was measured in dimethylformamide (DMF) solvent at ambient temperature and in the range of 200–800 nm by a JASCO-V570 spectrophotometer instrument. ^1^H and ^13^C NMR spectra were recorded in DMSO-d_6_ (deuterated dimethylsulfoxide) as solvent and tetra methylsilane as internal standard using a Brucker DPX FT/NMR-300 MHz spectrometer.

Fluorescence measurements were performed using a NanoLog modular (Horiba) spectrofluorometer equipped with glass cuvettes (1 cm path length). Emission spectra were recorded under excitation with a Xenon lamp at a fixed wavelength of excitation (λ: 340 nm) and slit 9. L solution was prepared as described above and the addition of zinc ion was carried out directly in the solution. Moreover, changes in fluorescence intensities vs. time appear immediately (and after 15 min). However, the fluorescence intensity of L decreases immediately and does not undergo any variation in the following times, demonstrating that the complex with the zinc ion is formed.

### 2.4. Electrochemical Tests

Electrochemical measurements were performed using a commercial SPCE, comprising a planar substrate equipped with a 4 mm carbon working-electrode (geometric area, 0.1257 cm^2^), a silver pseudo-reference electrode and a carbon auxiliary electrode. Cyclic voltammetry (CV) and potentiometric measurements were performed using a DropSens µStat 400 Potentiostat powered by Dropview 8400 software for data acquisition.

Bare screen-printed carbon electrodes (SPCEs) were easily modified as follows. A quantity of 1.0 mg of L was ultrasonically dissolved in 0.5 mL of DMSO. Thereafter, 2 µL of the solution was directly dropped onto the surface of the carbon working electrode and were allowed to dry at room temperature until further use.

Moreover, a L-M/SPCE sensor was obtained by mixing the Schiff base ligand with a conductive membrane (M). The membrane was prepared by mixing 0.5 mg of sodium tetraphenylborate (NaTBP), 33 mg of polyvinylchloride (PVC), 65.71 mg of 2-nitrophenyloctylether (2-NPOE) and 1 mg of L. The components were dissolved in 1.0 mL of THF and 2 µL of the mixture was deposited on the sensor. The solvent was allowed to dry overnight at room temperature to obtain a transparent membrane.

Linear sweep voltammetry (LSV) and potentiometric calibration curves using SPCE were performed by adding small volumes of the metal ions stock standard solutions into the electrochemical cell containing 3 mL of distilled water. Three scans were made for each new unit in buffer solution before taking reliable measurements to stabilize the SPCEs. To ensure the elimination of the metal ions from the working SPCE surface, a conditioning potential at 1.3 V for 30 s was applied before each measurement. All experiments were carried out without any oxygen elimination and at room temperature (25 °C).

## 3. Results

### 3.1. Characterization of Schiff Base Ligand

**Scheme 1:**
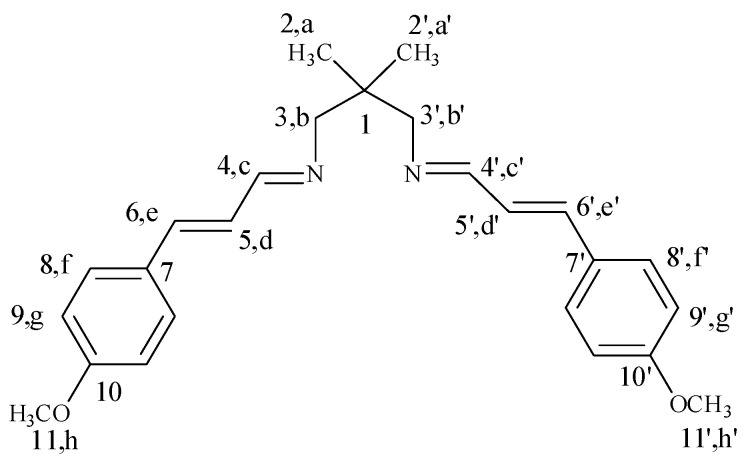
Atom numbering representation of Schiff base ligand L.

A schematic representation of the L ligand is shown in Scheme 1. Data related to FT-IR, NMR, UV-Vis spectra and molar conductivities of Schiff base ligand L were described in previous Section 2.

**Figure 1:**
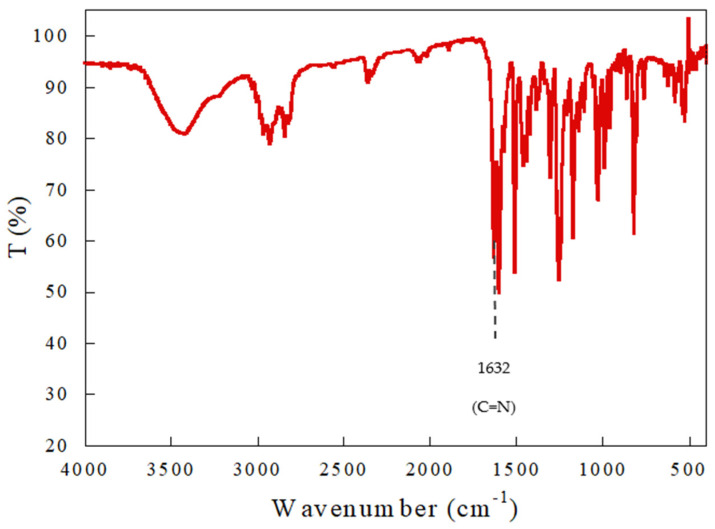
FT/IR spectrum of ligand L.

FT-IR spectroscopy is the first and easiest step to identify novel synthetic compounds via functional groups. Figure 1 shows the IR spectrum of the Schiff base ligand L. Compared to the expected bands related to reactants, the lack of stretching vibrations of NH_2_ of primary amine at 3285 cm^−1^ and C = O of 4-methoxycinnamaldehade at 1685 cm^−1^ as initial reactants, and the appearance of a new sharp and strong peak at 1632 cm^−1^ indicating formation of azomethine or iminic (C = N) bond as a characteristic functional agent of the Schiff base compounds, support the idea that the titled Schiff base ligand was successfully synthesized. Moreover, the weak vibrations at 3058 and 2965 cm^−1^ are assigned to the stretching vibrations of C-H bonds of aromatic and aliphatic fragments, respectively. Further bands between 2900 and 2800 cm^−1^ are associated with the stretching vibrations of the aliphatic C-H groups, whereas C-H bending of the methyl group appears at 1400 cm^−1^. Finally, the strong band at 1275–1200 cm^−1^ is due to C-O stretching of aryl ether.

**Figure 2:**
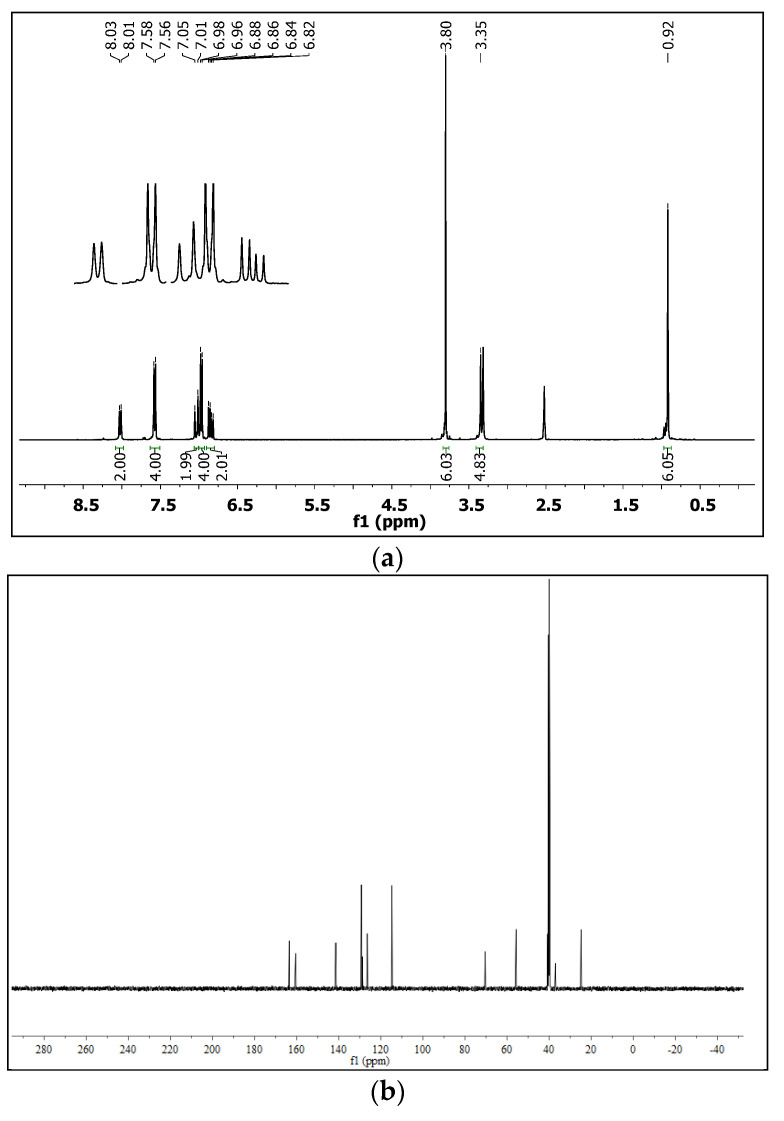
(**a**) ^1^H NMR spectra of ligand (L) and (**b**) 13C NMR spectra of ligand L.

^1^H NMR and ^13^C NMR spectra of ligand(L) based on Scheme 1 are exhibited in Figure 2. In the ^1^H NMR spectrum of the ligand, azomethine hydrogens of **cc′** are observed at 8.02 ppm as a doublet signal with coupling constant of 8.7 Hz. Olefinic hydrogens of **ee′** and **dd**′ in the ligand spectrum appear at 7.03 and 6.85 ppm as doublet and doublet of doublet, respectively. Aromatic hydrogens of **gg′** and **ff′** in the ligand spectrum are seen at 6.97 and 7.57 ppm as doublet peaks. Aliphatic hydrogens of **aa′**, **bb′** and **hh′** in the ligand appear at 0.92, 3.35 and 3.80 ppm as singlet peaks, respectively. In the ^13^C NMR spectrum of ligand L, the characteristic signal is related to azomethine carbon appearing at 163.57 ppm. Aromatic, olefinic and aliphatic carbons of ligand L appear at 126.42–160.48, 114.76–141.46 and 24.79–70.40 ppm, respectively.

**Figure 3:**
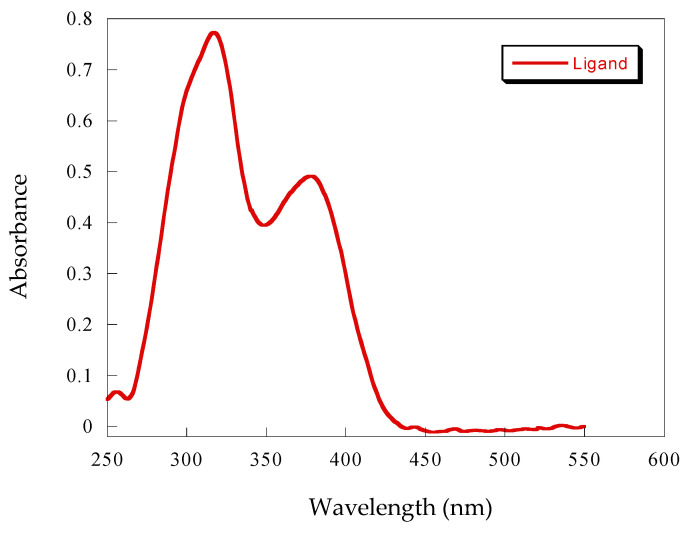
UV-Vis spectrum of the Schiff base ligand L.

Electronic spectrum of L in the DMF solution (10^−5^ M) was recorded in the range of 200–800 nm at room temperature and the relevant information is found in Figure 3. In the UV– visible spectrum of the ligand, two intense absorption bands were observed. The first band appeared at 318 nm and is attributed to π→π* electronic transitions of the ligand (aromatic rings and alkene moiety). The second absorption band of the ligand at 380 nm may be attributed to n→π*/π→π* of imine groups.

### 3.2. Fluorescence Studies

**Figure 4:**
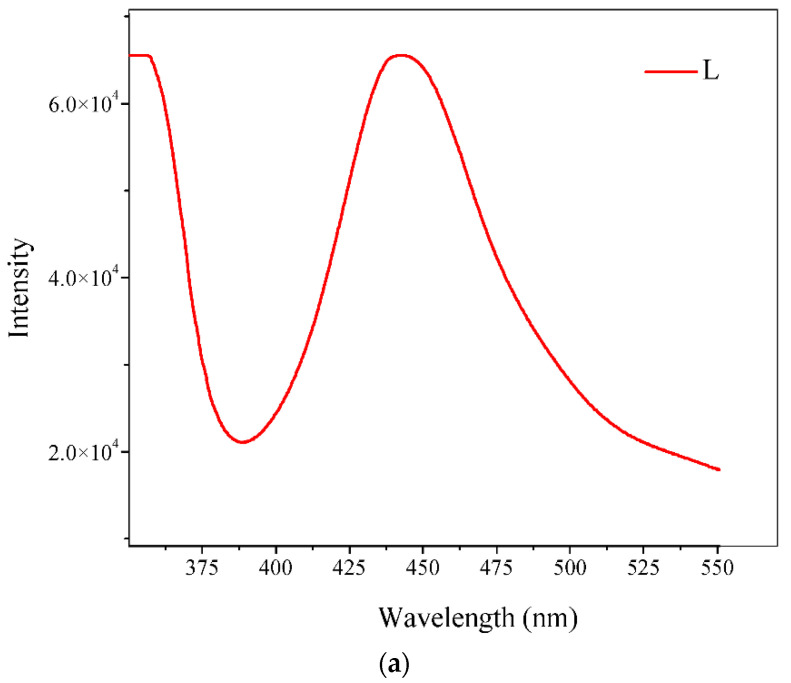

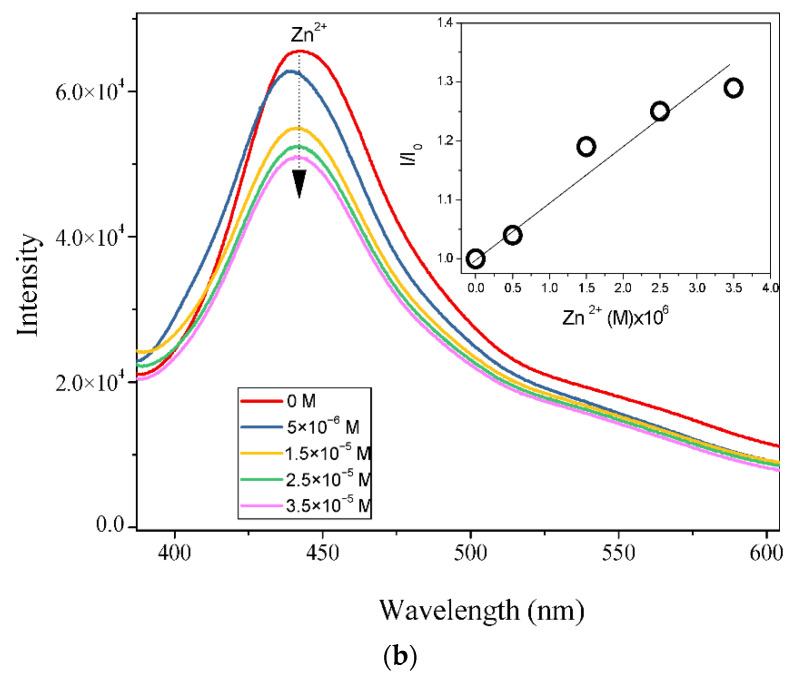
(**a**) Photoluminescence emission peak of the Schiff base ligand L; (**b**) variation in the photoluminescence peak at the addition of different Zn ions concentrations from 0 to 3.5 × 10^−5^ M. Inset shows the fluorescence quenching Stern–Volmer plot with increasing concentration of Zn ions.

Schiff bases are known to exhibit fluorescence characteristics in both solution and a solid state. The fluorescence from L in the visible region (see Figure 4a) can be explained by the presence of the extended conjugation. At the excitation length of 340 nm, the base shows a fluorescent emission behavior with a well-defined band at 435 nm.

Schiff bases display versatile coordination chemistry with transition metal ions due to the preferred coordinative interaction with the imine –N and –OH groups; therefore, in recent years, Schiff bases have been investigated for the detection of various toxic metal ions [20,21]. Depending on the ligands and the metal ion, fluorescence tuning both in intensity and/or emission maximum can be observed. A fluorescence enhancement [22] or reduction (fluorescence quenching [23]) can occur upon metal ion coordination.

**Scheme 2:**
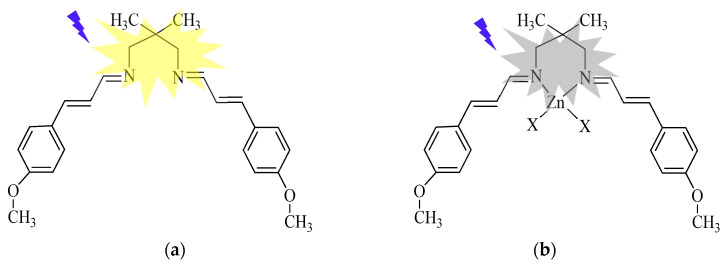
Schematization of the fluorescent emission behavior for: (**a**) ligand L; (**b**) Zn-L coordination complex. Wavelength excitation is 340 nm.

Investigating the PL properties of L, we found that the addition of Zn ions caused remarkable changes in the PL peak intensity (Figure 4b). The interaction between Zn ion and the L Schiff base is assumed to transfer energy to Zn(II) and hinder the ligand-centered emission, resulting in the luminescent quenching of L [24]. The inset in Figure 4b shows the analysis of data on the basis of the Stern–Volmer I/I_o_ = 1 + K_sv_[Zn^2+^] equation, where *I* and *I*_0_ are the fluorescence intensities of the L solution in the presence and absence of Zn ions, K_sv_ is the Stern–Volmer constant, and [Zn^2+^] the Zn ions concentration. The K_sv_ value was in the order of 10^4^–10^5^ M^−1^, suggesting a significant interaction of Zn ions with L, which leads to the fluorescence quenching. The quenching pattern observed was then due to the formation of a non-fluorescent Zn-L coordination complex (see Scheme 2).

### 3.3. Electrochemical Studies

**Figure 5:**
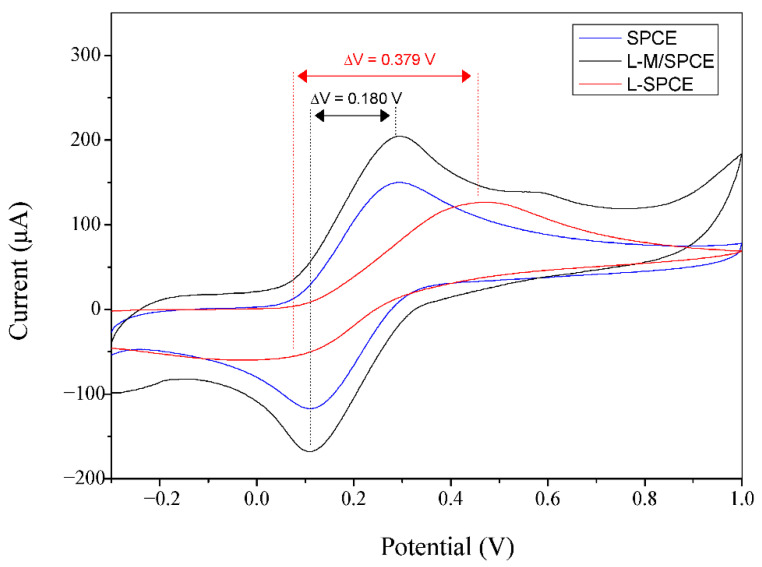
CV measurements of SPCE, L-SPCE and L-M/SPCE in PBS solution; CV of 10 mM (Fe(CN)_6_^3−/4−^) solution at a scan rate of 50 mV s^−1^.

Based on the promising coordination ability for Zn ions, the L ligand was used to modify a commercial screen-printed electrode for developing a simple electrochemical device for the electroanalytical determination of these ions. First, the electrochemical properties related to the modified sensors were evaluated by cyclic voltammetry (CV). CV measurements were recorded in 1.0 mM phosphate buffered saline (PBS) solution at pH 7.4 as electrolyte and in the presence of the ferro/ferrocyanide (Fe(CN)_6_^3−/4−^) redox couple. Cyclic voltammograms were recorded in 10 mM (Fe(CN)_6_^3−/4−^) solution at a scan rate of 50 mV s^−1^. For all the bare and modified SPCEs, a pair of distinct, well-defined redox couple peaks were observed between −0.3 and +1 V (Figure 5).

On the bare SPCE, a pair of redox peaks with the peak-to-peak separation (ΔEp) of 0.180 V was observed. On the L/SPCE, redox peaks are largely suppressed, likely due to the insulating layer formed after the Schiff base deposition on the working layer. Further, the (ΔEp) was extended to 0.379 due to the presence of ligand L, which limits the diffusion process. On the L-M/SPCE, a higher peak current was observed in comparison to SPCE and L/SPCE. The presence of the membrane restores the electrochemical properties of the L-M based electrode, which could be ascribed to the high ionic conductivity of the M membrane used together with the L ligand.

The effective electrochemical active surface area of the prepared electrode was also determined from these data based on the Randlese–Sevcik equation. The formula was employed to determine the active surface area of the SPCE and L-M/SPCE using the ferro/ferrocyanide as the redox probe:

$$\mathrm{Ip}=\pm 0.4463\mathrm{nFAC}\surd \frac{\mathrm{nFvD}}{\mathrm{RT}}$$

where Ip is the voltammetric current (A) using the forward peak of the electrochemical process, n = 1 is the number of electrons transferred in the electrochemical reaction, F = 96485.3365 C/mol is the Faraday constant, A is the electrode surface area (cm^2^), C = 10^−5^ M/cm^3^ is the concentration of the redox probe, v = 0.05 V/s is the applied voltammetric scan rate, D = 7.6 × 10^−6^ cm^2^/s is the analytic diffusion coefficient, R = 8.314 J/mol is the universal gas constant and T = 298.15 K is the Kelvin temperature. The increase in the effective electrochemical active surface area of L-M/SPCE was about 16% compared to the bare SPCE.

**Figure 6:**
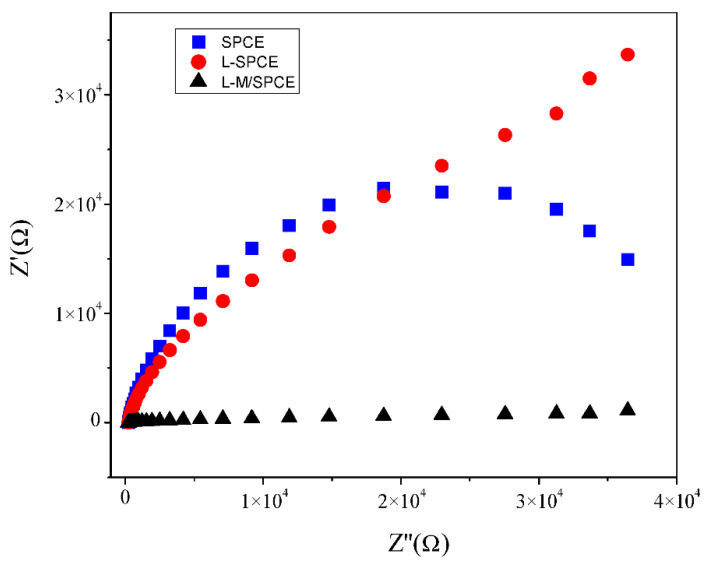
EIS of the fabricated sensors in 10 mM (Fe(CN)_6_^3−/4−^) solution.

Further investigation of the electron transfer properties at the bare and modified electrodes was performed using electrochemical impedance spectroscopy (EIS). Here, the changes in the impedance and interface properties during the electrode surface modification procedure were measured and are shown in Figure 6.

EIS experiments were carried out in a 10 mM (Fe(CN)_6_^3−/4−^) solution over the 0.1–100,000 Hz range. Typically, impedance spectra consist of semi-circular and linear regions that represent electron transfer and diffusion processes, respectively. Electron transfer processes occur at higher frequencies, whereas diffusion processes occur at lower frequencies. The results reported highlight that the L-M layer causes a notable decrease in the impedance with respect to bare SPCE and L/SPCE, confirming the beneficial role of an ionic membrane used in cooperation with Schiff base ligand. These enhanced electrochemical properties encouraged us to investigate the developed L-M/SPCE as a sensor for the analytical determination of metal ions, in particular Zn (II).

### 3.4. Electroanalytical Applications

**Figure 7:**
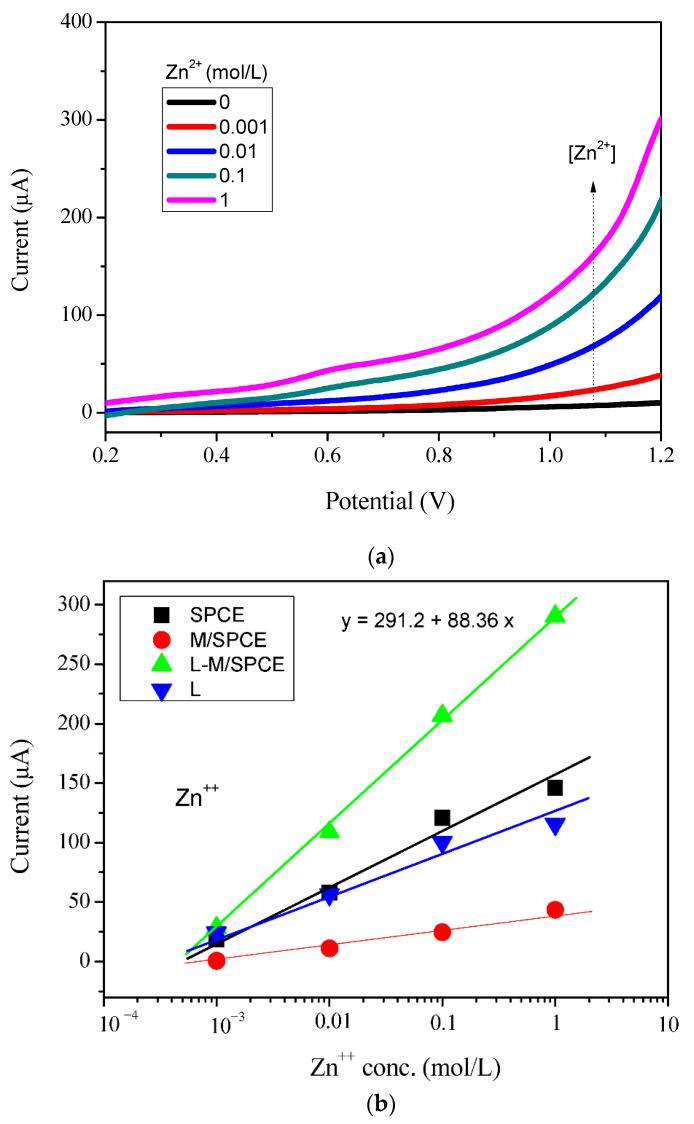
(**a**) I-V tests carried out on L-M modified SPCE in the presence of different concentrations of zinc ion. (**b**) Current registered at 1.2 V vs. concentration of Zn ions in solution for the various fabricated sensors.

For the analytical application of the fabricated electrochemical sensors, several voltammetric methods can be applied [25]. To investigate the capabilities of bare and modified SPCE electrodes for the detection of Zn ions in distilled water, the LSV method was first applied. The curves shown in the graph in Figure 7a report the current of the L-M/SPCE sensor subjected to an increase in the potential from 0.25 to 0.90 V in the presence of different concentrations of Zn ions in solution. The registered current increases contemporaneously with the applied potential and concentrations of Zn ions.

Plotting the current registered at the potential of 1.2 V, we observe a good linearity vs. zinc ions concentration in the range of 10^−3^–1 M (Figure 7b). From the statistical analysis applied, the equation found was y = 291.2 + 88.36 x, and R^2^ = 0.99798. In this figure are also reported the data collected in the same conditions for the bare SPCE, M/SPCE and L/SPCE sensors. A different response of the investigated sensor to Zn^2+^ is clearly noted. The response of the L-M/SPCE sensor was higher than the response of the other sensors tested.

**Figure 8:**
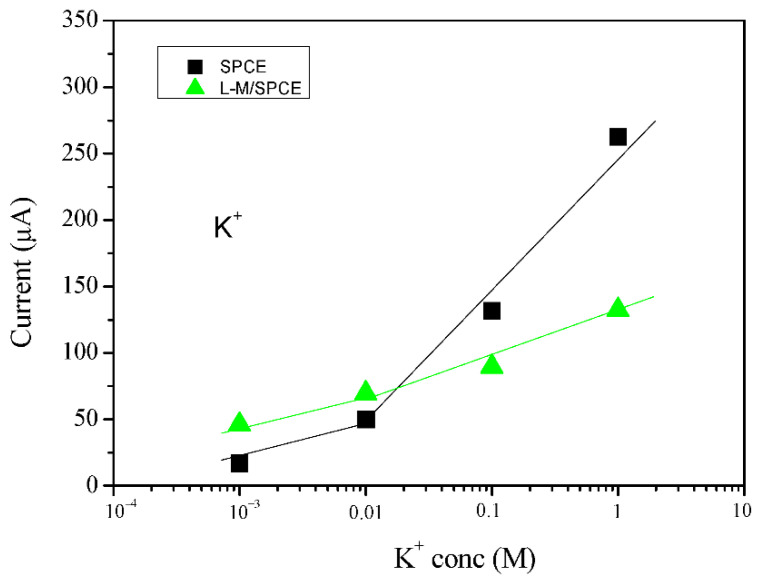
Current registered on bare SPCE and L-M/SPCE sensors at 1.2 V vs. concentration of K ions in solution.

Results from the LSV method demonstrate that the modification of the bare SPCE electrode with the combination of L as the ionophore and M as the ionic membrane contributes to the improved electrochemical detection of zinc ions compared to bare SPCE. As support for this, we present in Figure 8 data obtained with K ions. This ion was chosen because it is not coordinated at the modified L-M/SPCE electrode. We can observe on bare SPCE and L-M-SPCE sensors a different response in solutions containing K ions compared to the behavior reported in Figure 7 in the presence of Zn ions, confirming that the characteristics of the L-M/SPCE sensor are linked to the coordination ability towards Zn ions.

Potentiometry is one of the most commonly developed electrochemical methods, and is based on the measurement of a potential difference when the target ionic species in an aqueous phase complex with target selective ionophores present in an organic membrane [26]. Potentiometric sensing can be an attractive alternative to the existing electrochemical analytical tools due to its simplicity and fast responses [27]. Figure 8 shows the selective response of the bare SPCE and L-M/SPCE sensors to zinc ion addition. It can be observed that the response of the modified electrode is highly amplified with respect to the bare electrode with a sensitivity of 50.1 mV/dec.

**Figure 9:**
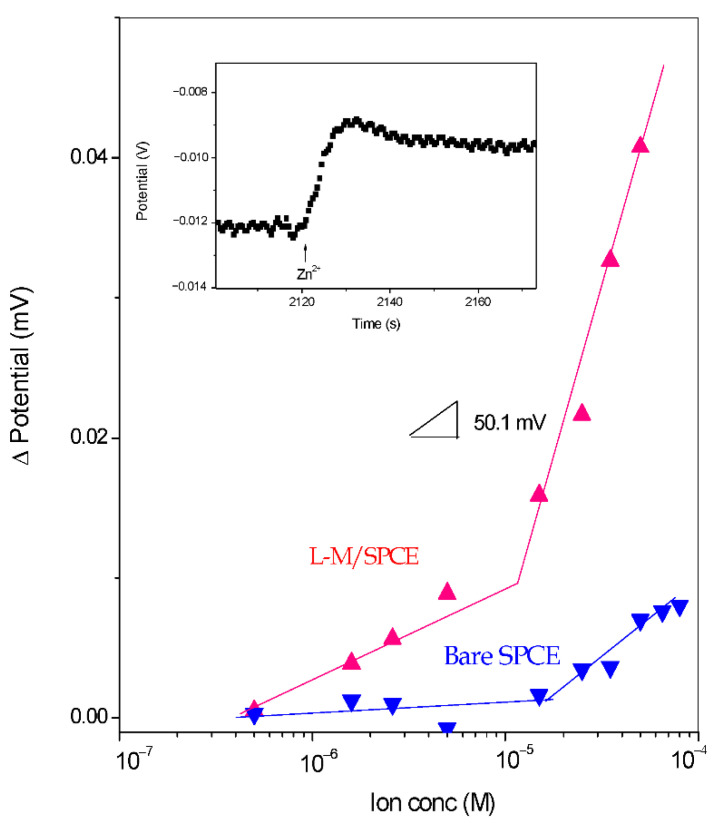
Calibration curves for the SPCE and L-M/SPCE sensors to different concentrations of Zn ions. Dynamic response of the L-M/SPCE sensor for a step change in the concentration of Zn^2+^ ion.

The dynamic response time of the L-M/SPCE sensor was also evaluated. The inset in Figure 9 shows the potential variation of the sensor to a step change in the concentration of Zn^2+^ ion. A fast response was noted; indeed, the time required for the Zn^2+^ sensor to reach the new potential value was about 10 s.

**Figure 10:**
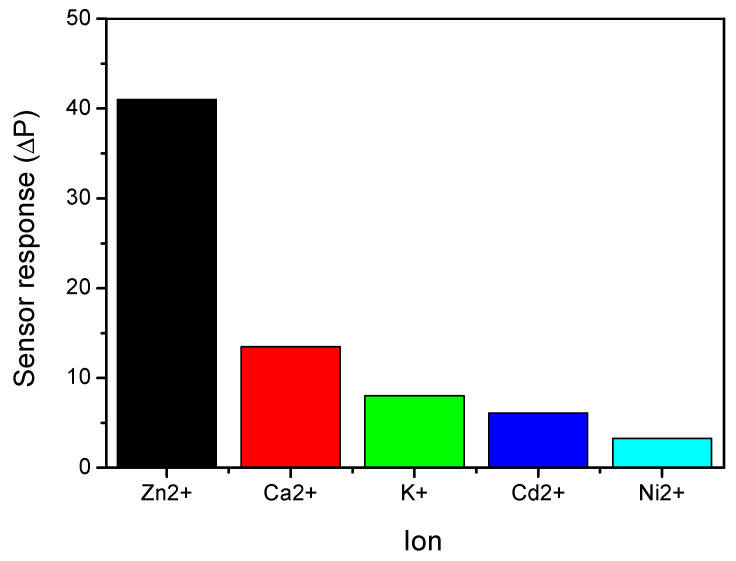
Response of the L-M/SPCE sensor to different ions. The concentration of the ions is equal to 5.5 × 10^−5^ mol/L.

Aqueous solutions of both monovalent and divalent cations were also added, measuring the potential changes for each addition, for evaluating the selectivity of the sensor. The concentration was kept the same for all cations (5.5 × 10^−5^ mol/L) and the additions were made directly to the solution, which was kept under mechanical stirring. According to the potentiometric results, the sensitivity towards Zn ions of the proposed sensor is much higher compared to other ions, thus evidencing its good selectivity (Figure 10). Regarding the LSV method, the high selectivity registered with the potentiometric method is due to the high affinity of the L-M/SPCE sensor for Zn compared to the other metal ions.

To explain the observed behavior, we believe that the sensor response is generated by: (i) the electric double layer at the interface between the solution and (ii) the L-M membrane and the ion transfer from the solution to the L-M membrane. That is, the observed response is the result of the combined effects of the ionic conductivity of the ion solution and the resistance at the electrode/ion solution interface. At a certain concentration of Zn^2+^, the first contribution is constant, so the electrode/ion solution interface resistance is the dominant factor, and this agrees with the different EIS spectra obtained on the various electrodes. However, the highest response of the L-M/SPCE potentiometric sensor can be explained by the preferential adsorption of Zn^2+^ onto the electrode surface. Indeed, as demonstrated by a photoluminescence study, nitrogen as a supplier of donor atoms in the L ligand acts as a strong coordination site for binding zinc, enhancing the electrochemical properties of the L-M/SPCE membrane towards the detection of zinc ions.

Table shows a comparison of the Zn^2+^ sensing performances of our electrochemical sensor with others reported in the literature. As is well known, electrochemical techniques based mainly on stripping voltammetry (ASV) methods have been widely reported in the scientific literature for Zn ion detection because of their high sensitivity [15,16,17,18]. These electrochemical sensors undoubtedly have high performance, but the presence of some toxic components (especially in the mercury-based electrodes) limits their acceptance in wide-scale routine analysis. Therefore, the potentiometric Zn sensor based on the novel synthesized Schiff base L may be a strong competitor, with similar or better electrochemical properties, compared to sensors with other Schiff bases or based on different electrode sensing materials proposed to date (see refs. [28,29,30,31] in Table 1). In addition to compliance with safety regulations, this sensor has the advantage of enabling a simple, cost-effective, and easy preparation process.

The issue of selectivity is also of particular importance for the practical applicability of these sensors. Most metal ions, in particular Ca^2+^, are usually present in high concentrations in water samples and can interfere in the detection of zinc ions. Very selective modified electrodes with a Schiff base, such as SMS-3, having high affinity toward Zn ions, have been proposed [28]. Other Schiff bases have been reported in the literature for developing high-performance electrochemical/fluorescent sensors for Zn ions, but the selectivity issue is not addressed (see refs. [29,32]).

Schiff bases are also used as fluorescent probes for the detection of zinc ions [32,33,34]. Obviously, this technique displays better performance regarding the selectivity issue, but needs more complicated instruments than the electrochemical approach. Instead, the proposed electrochemical sensor for Zn ions is of low cost and simple to use. In the case of the presence of high concentrations of interferent ions such as Ca^2+^, a selective preconcentration step must be applied to overcome this problem [35]. In contrast, in the determination of Zn in foods and pharmaceutical tablets, the preconcentration step is not necessary [36,37].

## 4. Conclusions

In conclusion, the novel synthesized Schiff base L was found to be suitable as a sensing material for the detection of Zn ions in water. Photoluminescence studies showed that L displays a fluorescence turn-off in the presence of Zn2+ due to its high coordination ability for this ion. A simple electrochemical sensor was then fabricated by modification of SPCEs with L as the recognition layer, and showed a micromolar detection limit and good linearity with a high sensitivity of ∼50.1 mV/dec for sensing Zn^+2^ ions. A fast response time of about 10 s with good selectivity to some common interferent ions was also demonstrated. Further, these sensors are easy to fabricate and are intrinsically safe as compared to the traditionally used mercury-based electrodes.

Based on the knowledge acquired in this first study, our future work will focus on developing novel probes for heavy metal ions and the fabrication of electrochemical sensors based on these receptors.

## Figures and Tables

**Table 1:** Comparison of the Zn^2+^ sensing performances of sensors reported in the literature.

Sensing Material	Linear Range	Limit of Detection (LOD)	Ref.
Ionophore (SMS-3) Schiff base moiety modified SPCE	0.47 to 5.56 μM	0.92 µM	[28]
Aminoacid Schiff base modified GCE	10 to 150 µM	-	[29]
3-D printed carbon nanofiber–graphite–polystyrene	12.7 μg/L to 450 μg/L	8.6 μg/L	[30]
Screen printed bismuth oxide	75 to 600 mg/L	33 mg/L	[31]
Coumarin-based fluorescent sensor	100 pM to 1 mM	3 µM	[32]
L-SPCE	1 µM to 100 mM	3.5 µM	This work

## Data Availability

Not applicable.
